# Si APD-Based High Speed Infrared Radiation Thermometry for Analysing the Temperature Instability of a Combustion Chamber

**DOI:** 10.3390/s24237780

**Published:** 2024-12-05

**Authors:** Louis Karapateas, Yufeng Lai, Xiangfei Meng, Yang Zhang, Jon R. Willmott, Matthew J. Hobbs

**Affiliations:** 1 Sensor Systems Group, School of Electrical & Electronic Engineering, The University of Sheffield, Portobello Centre, Pitt Street, Sheffield S1 4ET, UK; lkarapateas2@sheffield.ac.uk (L.K.); y.lai@sheffield.ac.uk (Y.L.); j.r.willmott@sheffield.ac.uk (J.R.W.); 2 School of Mechanical, Aerospace and Civil Engineering, The University of Sheffield, Sir Frederick Mappin Building, Sheffield S1 3JD, UK; omeng1@sheffield.ac.uk (X.M.); yz100@sheffield.ac.uk (Y.Z.)

**Keywords:** avalanche photodiode, droplet combustion, ignition, infrared radiation thermometry, field-programmable gate array

## Abstract

This study introduces a novel approach to analysing the combustion process using a high-speed, non-contact, optical fibre-coupled Si avalanche photodiode (APD)-based infrared radiation thermometer (IRT). The Si APD-IRT, combined with an optimised field-programmable gate array (FPGA)-based digital design, achieves a response time of 1 µs, faster than commercially available instruments. Our instrument captures the entire ignition and reignition cycle of a Jet A kerosene droplet with high temporal precision within a combustion chamber, a feat impossible with traditional thermocouples. The FPGA module was validated with a 1 µs data acquisition time, using a 40 MHz onboard clock, achieving throughput of 0.64 Gbps with efficiencies of 0.062 Mbps/slice in lookup tables (LUTs), confirming a low-area design compared to conventional FPGAs. The IRT achieves a root mean square (RMS) noise specification of 0.5 °C at a 1 µs acquisition time and a target temperature of approximately 1000 °C. A measurement uncertainty of within ±0.25% °C + 2 °C confirms that it lies within the bounds of commercial instrumentations. Our instrument was demonstrated to capture transient temperature fluctuations during combustion and characterises Jet A kerosene fuel droplets, laying the foundation for understanding sustainable aviation fuels (SAFs) and their role in transitioning from aviation fossil fuels, enabling effective research and development.

## 1. Introduction

The American and European air safety regulations require all aircraft to successfully carry out high-altitude reignition after flameout [1]; the characteristics of this flameout are directly related to the properties of the fuel used. Fossil fuel-based aviation fuel, or jet fuel, contains specific additives that ensure its stability and reignition under harsh high-altitude conditions, enhancing performance under extreme temperatures and altitudes by improving lubricity, preventing corrosion, and enhancing thermal stability. Its properties and therefore its behaviour when reignited are well understood. However, to mitigate the heavy dependence on fossil fuel-based aviation fuel, ultimately reducing the carbon footprint of the aviation industry, sustainable aviation fuels (SAFs) are being developed [2]. Therefore, to understand the reignition process for future utilisation, it is essential that combustion studies for SAFs are carried out to enable the same depth of understanding as those for traditional fossil fuel-based aviation fuel under the aforementioned conditions [3,4].

Typical studies on aviation fuel use a controlled combustion chamber to simulate environmental and relight conditions. Due to the complexity of modelling spray combustion under these conditions, single-droplet combustion studies effectively help the understanding of the overall spray combustion mechanisms [5,6]. An analysis of the closed combustion chamber properties reveals temperature fluctuations caused by droplet ignition and the gas-phase flame. Effective diagnostics and maintenance are crucial for the reliable operation of systems like ramjet combustors. Forced ignition under high-altitude reignition conditions is being studied to enhance aeroengine safety and efficiency. Additionally, studying liquid fuel combustion at low pressures is essential for enhancing stability and reducing ignition delay [7,8].

One of the key challenges in characterising droplet characteristics is the inherent high-speed transients associated with various scales of micro-explosion typical of those that are representative of aircraft cruise speed. These micro-explosions result in rapid changes in system temperature, highlighting the critical need for high-speed measurement instrumentation in order to better understand the combustion process. Accurate measurements are essential for capturing temperature fluctuations and gaining an understanding of the scale of temperature instabilities that can be characterised within a closed chamber during combustion.

Traditional thermocouple-based instrumentation, typically used for temperature measurement in combustion applications, can introduce inaccuracies due to physical contact. This can disrupt the droplet’s temperature through heat transfer, resulting in the failure to capture the surrounding environment’s impact and flame propagation [9,10]. Examples like indium tin oxide (ITO) thin film and K-type thermocouples often disrupt the droplet’s temperature, resulting in inaccurate temperature measurements. They primarily capture the droplet’s surface temperature and have a limited ability to reflect flame distribution, making them unsuitable for accurately measuring the gas-phase flame in combustion [11]. Thermocouples are also inherently slow due to their contact measurement nature, with response times around 10–20 ms [12,13]. They are not suitable for measuring fast transient temperature changes, leading to incorrect conclusions regarding temperature changes within closed chambers during combustion. Given that transient changes are expected in the order of microseconds, a significantly faster temperature measurement solution is required. Current high-speed solutions, such as non-intrusive laser techniques like Planar Laser-Induced Fluorescence (PLIF) and Coherent Anti-Stokes Raman Spectroscopy (CARS), are expensive, complex to operate, and used to selectively detect specific temperature regions within combustion processes [14]. However, such methods do not provide a comprehensive view of the entire combustion process, despite their high-speed capabilities.

In comparison, non-contact infrared radiation thermometers (IRTs) are ideal for high-speed measurement applications, providing quick and accurate temperature readings without the requirement for physical contact [15]. Typical commercial instruments advertise response times as low as 1 ms, although some instruments offer an analogue output in as little as 6 µs, albeit without digital acquisition [16]. Inherently high-speed applications that benefit from such a measurement approach include confined blast measurements, ballistics, and the monitoring of rotating turbine blades in power plants and jet engine tests [17,18,19,20]. High-speed, non-contact IRTs are therefore the ideal solution to perform investigations on the thermal characteristics of the fast transient temperature changes expected within micro-explosions.

In the process of designing an IRT capable of high-speed measurements within such high-speed applications, there are several considerations required to complete optimisation. The use of an avalanche photodiode (APD) instead of a standard photodiode is one such consideration due to its enhanced sensitivity. This design choice is highly advantageous to the performance of high-speed IRTs, enabling faster measurements and the detection of weaker signals. This ultimately enables the measurement of lower target temperatures whilst still satisfying the noise specification typical of instruments operating at the comparative wavelength [21,22,23,24]. In terms of digital acquisition, there are also important considerations to be made. In recent high-speed Internet of Things (IoT) applications, the use of a data acquisition (DAQ) unit with an embedded Field Programmable Gate Array (FPGA) is often utilised to achieve an increase in the operating clock frequencies and device efficiency, resulting in higher throughput and acquisition rates [25]. This offers a significant advantage over conventional microcontrollers [26] and DAQ units, such as the USB-6212, which uses slower ADCs to process data at speeds down to 10 µs. However, such approaches can cause delays due to sequential processing, especially when logging high-speed data [27].

Furthermore, the integration of a Direct Memory Access (DMA) unit and an ADC unit within FPGAs can greatly enhance the resolution of the data captured. This enhancement not only increases the level of detail and clarity in the measurements, but also significantly improves instrument accuracy. FPGAs are inherently customisable, allowing for further optimisation within the digital electronics. Such advancements can lead to the development of a digital data acquisition system that is well suited for high-speed measurements, such as those required to enable high-speed IRT operation.

Other high-speed solutions incorporate the use of Compact RIO (cRIO) systems, which consist of a real-time controller, an FPGA, and I/O modules. These components facilitate communication between the FPGA and external devices, handle high-speed data processing, and execute complex algorithms [28]. This is possible due to the cRIO’s low-latency data handling optimisation, achieving data acquisition speeds up to 4 µs [19,29,30]. Some approaches within cRIOs utilise higher synthesised user-controlled I/O functions, which are even faster due to the FPGA module’s direct processing capabilities. This enables parallel processing, simultaneously allowing multiple operations and ensuring lower latency, resulting in a documented 1 µs data acquisition time.

In this work, we developed and deployed a high-speed non-contact IRT for measuring the temperature inside a sealed chamber during both the droplet ignition and the subsequent gas-phase flame of standard aviation Jet A kerosene fuel. Utilising a highly sensitive Si APD and an embedded high-speed FPGA-based DAQ system, we measured and evaluated the early stages of droplet ignition at a data acquisition time of 1 μs. This was achieved using an NI 9030 cRIO and NI 9223 module, which required a digital design in NI LabView 2019 32-bit (National Instrument Corporation, Austin, TX, USA), a process that involves the Reset I/O function, interrupt signalling to the FPGA, the Generate I/O Sample Pulse function, and a DMA First In First Out (FIFO) module. Our approach enables the examination of rapid transient temperature fluctuations caused by micro-explosions in a closed chamber [31,32]. The high-speed instrument detects temperature transients, leading to combustion chamber instabilities, which are crucial for observing electric sparks and flames throughout the combustion process and previously unattainable with contact solutions. To the best of our knowledge, this is the first measurement of its kind within combustion applications that captures such fast transient temperature measurements. These high-speed measurements enhance data precision within the characterisation of the combustion process. This will provide a valuable future tool for the characterisation and development of SAF as a potential replacement for fossil-based fuels in aviation, contributing to a more sustainable future.

## 2. Materials and Methods

### 2.1. Experimental Overview and Methods

**Figure 1:**
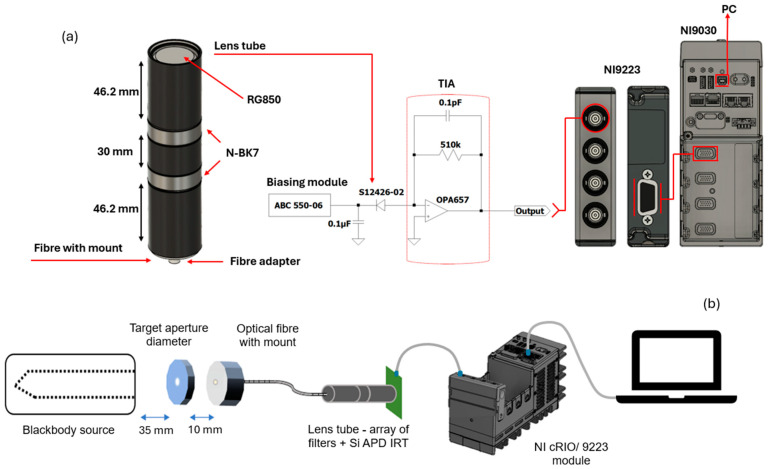
(**a**) Detailed schematic representation demonstrating the connections between various components and (**b**) visual representation of the experimental setup.

The IRT used in this work was derived from a Hamamatsu (Hamamatsu Photonics K.K, Hamamatsu City, Japan) S12426-02 Si APD and consisted of a transimpedance amplifier (TIA) circuit with a theoretical time constant of 51 ns (R = 510 kΩ and C = 0.1 pF), as shown in Figure 1a. An APD was chosen over a photodiode, which is advantageous for this high-speed application due to its high internal gain. The specific APD used within this work was chosen due to its lower operating bias voltage and a longer wavelength response compared to alternative Si APDs.

The circuit did not incorporate any further RC filtering beyond this in order to maximise the response time of the analogue electronics. An NI cRIO 9030 FPGA (National Instrument Corporation, Austin, TX, USA) equipped with a high-speed NI 9223 module utilising a 16-bit FPGA design, was used to capture the analogue output of the IRT, with high-speed sampling capabilities up to 1 MS/s per channel. The module includes 8 MB of memory storage per channel, enabling faster parallel operations and allowing flexibility for significant real-time software changes through LabView, making it ideal for high-speed applications. This resulted in an overall IRT-DAQ instrument with an acquisition time of 1 μs. To achieve this, the cRIO was configured based on the scalability of the controlled I/O sampling method in LabView, a method that uses a continuous looping sequence that generates a pulsing, reading, and writing sequence within the DMA FIFO module.

The APD was optically coupled to a silica fibre optic assembly consisting of single N-BK7 Plano-Convex Lenses (LA1131-ML) from Thorlabs, Inc (Newton, NJ, USA). The system did not incorporate any further field or aperture stops, though an RG850 daylight filter was also incorporated into the design from Thorlabs, Inc (Newton, NJ, USA). The fibre was sighted in front of a target aperture placed in front of an Ametek Land R1500T blackbody calibration furnace (Dronfield, UK), as shown in Figure 1b, to enable radiometric measurements to take place.

### 2.2. Instrument Calibration

Before performing calibration, the output voltage of the IRT was measured as a function of target temperature, ranging from 600 °C to 1000 °C. To maintain traceability to ITS-90 [33], the radiance temperature of the furnace was measured using a calibrated Ametek Land Cyclops C100L IRT (Dronfield, UK) [34,35]. The radiometric calibration of the instrument was based on Planck’s law [36], as shown in Equation (1). This equation represents the spectral radiance of a black body source (*L*) as a function of temperature (*T*) in Kelvin and wavelength (λ). It incorporates the first and second radiation constants, *c*_1_ and *c*_2_, and the bandwidth of the integral between the cut-on and cut-off wavelengths *λ*_1_ and λ_2_, respectively. These wavelengths are derived from the RG850 filter and the cut-off wavelength of the Si APD, respectively. The photocurrent from the Si APD and consequently the output voltage of the IRT (*V*) are proportional to *L*. A five-point calibration was performed to fit the output voltage to Planck’s law, resulting in a calibration lookup table (LUT) mapping the relationship between the output voltage and temperature.

(1) $$L(\lambda ,T)=\frac{c_{1}}{\lambda^{5}}e^{\left(\frac{-c_{2}}{\lambda T}\right)}\mathrm{which}\;\mathrm{gives}\;V\;\mathrm{as}\;\int_{\lambda 1}^{\lambda 2}(\lambda )\;L(\lambda ,T)d\lambda$$

The field of view (FOV) of the IRT, measured to be a 3:1 target-to-source ratio, was established over varied diameter target apertures, with the instrument sighted upon this target aperture placed in front of the furnace. This setup ensures that the FOV encompasses the entire combustion chamber, as discussed in Section 2.4. The nominal FOV of the IRT was defined to represent 98% of the radiant power of the source with respect to the paraxial image of the field stop of the system [37]. The size-of-source effect (SSE), measured beyond the nominal FOV, was found to be negligible for the instrument.

The root mean square (RMS) noise for the IRT can be calculated with respect to the standard deviation of the output voltage after calibration to temperature; commercial IRTs typically specify a maximum acceptable RMS noise value of ±0.5 °C [19,23]. This noise was assessed by recording the output voltage from the IRT using the cRIO acquisition system at its raw 1 μs sampling time over a 1 s period. These data were then converted to temperature, in degrees Celsius, °C, with the standard deviation representing the RMS noise of the IRT at each temperature point. By applying various degrees of moving averaging filter to the raw data, we were also able to assess the noise corresponding to response times of 10 μs, 100 μs, 1 ms, and 10 ms in a similar manner.

To assess the response time of the instrument’s acquisition, as opposed to the FPGA’s timing, a 1 kHz pulsed square wave waveform from an Agilent Technologies Inc. 33210A waveform generator (Santa Clara, CA, USA) was used with an 870 nm LED. The final response time was defined within the 10–90% range of the rise time recorded.

### 2.3. FPGA Design and Performance

Computer architectures are chosen and optimised based on the processing needs of a particular algorithm. To reduce execution times and therefore optimise the algorithm, tasks are divided into sequences determined by data-dependent stages. A single-point measurement architecture, such as the one used in an IRT, employs nested loops that execute when data are available. Data synchronisation techniques, achieved using FIFO modules that store data in arrays, prevent execution overlap when data are available, with the FIFO technique on a cRIO storing one element set per stage [38,39].

In this context, efficient data handling and processing are crucial, necessitating the use of LUTs as configurable logic blocks within FPGAs. These components store truth tables, allowing direct mapping of input combinations to specific output values, and typically handle 4 to 6 inputs, with each LUT producing a single output. A memory unit stores the truth table, while a multiplexer selects the output, together implementing complex Boolean functions and optimising FPGA resource usage [40]. To further enhance FPGA performance, slices group multiple LUTs with additional higher-level building blocks, thereby organising the LUTs to provide structural hierarchy. Slices also facilitate the creation of both sequential memory-based and combinational logic-based circuits. Their efficient grouping enhances area utilisation, playing a critical role in achieving high system throughput. When designing FPGAs, minimising physical space through a low-area design is crucial, as efficient use of LUTs and slices reduces signal travel distance, enhancing performance, lowering power consumption, and enabling high-speed data processing [41].

User-controlled I/O sampling architectures output a sampling rate of 1 MS/s, which is faster than other digital architectures, such as I/O nodes, which can only achieve rates of 300 kS/s [42]. This architecture ensures higher throughput due to its faster communication with the I/O node. This ultimately results in a higher bandwidth, consequently leading to a higher number of samples being collected when used within an IRT. These data logging processes use nested loops for repetitive execution, enabling the handling of multiple data points simultaneously, which ensures high throughput and a low-area design by reducing the logic elements used. Loop A, which calls the I/O sample pulse function to generate a 1 µs pulse, defines the acquisition’s response time. Loop B ensures synchronisation between modules by calling the read I/O status function at the same rate, checking each sample’s status and preventing data overwriting. Loop C handles timeouts by creating a 25 ns delay (matching the FPGA’s 40 MHz maximum frequency) to ensure each sample is ready before writing into the DMA FIFO module, which serves as a high-speed register.

To calculate the throughput, the maximum clock frequency is rated at 40 MHz, corresponding to a 25 ns clock period. The data path width throughout the FPGA design module for the IRT consists of 16 bits, including several processing steps such as shift registers and DMA FIFO modules. A compilation summary includes details on how the design was translated into hardware, optimised, and prepared for loading onto the FPGA. It typically covers resource utilisation, such as the number of logic blocks, LUTs, and flip-flops used, timing analysis (e.g., whether the design meets the required clock speeds), and any errors or warnings encountered during the process. Equation (2) demonstrates the throughput in Gbps, whilst Equation (3) shows the FPGA efficiency in Mbps per slice. The efficiency is based on the number of LUTs and occupied slices [43].

(2) Throughput=Data path width×FPGA Maximum frequency 

(3) $$FPGA\;efficiency=\frac{Throughput}{Number\;of\;slices\;(LUT\;or\;occupied)}\;$$

To calibrate the timing of the FPGA, the response time of the cRIO was characterised using a two-stage calibration metric. This was established over a transmitted triggering signal from a calibrated oscilloscope (Keysight Technologies, Inc., Santa Rosa, CA, USA) and a 1 kHz sine wave test signal from the waveform generator. This secondary oscilloscope and the cRIO were configured to detect the first positive edge of the triggering signal, initiating the recording of the test sine wave signal from the waveform generator. The signal at the first positive rise in the data acquisition of the sine wave was represented as the output voltage (*Vout*) with respect to time. To prevent any overlap, the acquisition time of the oscilloscope was set at 5 μs, compared to the 1 μs of the cRIO.

### 2.4. Droplet Test Setup

For an accurate and representative assessment of single-droplet combustion under high-altitude reignition conditions, it is important to be able to independently adjust the temperature and pressure. Whilst the measurements within this work were conducted under room temperature and ambient pressure conditions, the temperature and pressure within this setup can be varied between 253 K and 293 K, and 20 kPa and 101 kPa, respectively. The measurement can be treated as if we were measuring an ideal blackbody emitter, implying that for a body in thermal equilibrium, the emissivity equals the absorptivity, which is fundamental in understanding blackbody radiation, as stated by Kirchhoff’s law [36,44]. It should also be noted that, due to the enclosed nature of the chamber, the emissivity can be considered to be 1 [45].

Although this combustion chamber provides an enclosed environment, it is insufficient for detonation combustion. The standard kerosene fuel in this study is in liquid form, with fuel vapour generated mainly during ignition and self-sustained combustion. In single-droplet combustion studies, the deployed droplet is relatively small compared to the surrounding enclosed volume inside the combustion chamber. Due to low environmental pressure and temperature settings, the fuel vapour and air cannot form a near-stoichiometric homogeneous mixture throughout the entire chamber. Consequently, the combustion process remains as deflagration, leading to a gradual increase in temperature and pressure [8,9].

**Figure 2:**
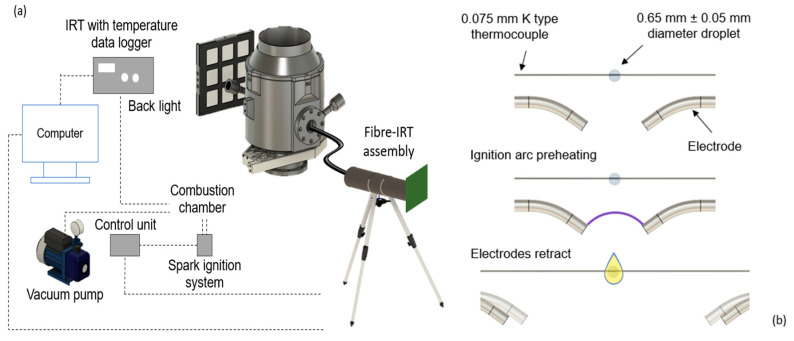
(**a**) Experimental setup of the combustion chamber and (**b**) droplet ignition dynamics.

The experimental setup, including the combustion chamber, is shown in Figure 2a. Inside the chamber, a single droplet of around 0.65 mm ± 0.05 mm in diameter of standard aviation Jet A kerosene was suspended on a type K reference thermocouple via a 10 μL micro-syringe pipette, as seen in Figure 2b, within a fixed position. This setup ensures that the droplet remains stationary throughout the combustion process, keeping the flame location relatively stable. Adjustments can be made to position the thermocouple wire, enabling the viewing window to capture the entire flame. The IRT, attached to the outside of the viewing window, records temperature changes caused by the flame. These measurements should remain consistent as long as the entire flame is visible and captured by the IRT. This positioning avoids cropping of any part of the flame, ensuring an accurate detection and precise temperature measurement within the combustion chamber, despite potential temperature variations.

Electric sparks were discharged from the ignition system and controlled via the control unit. Once the suspended droplet was ignited in the sealed combustion chamber, the combustion process was viewed by our instrument through a 25 mm diameter viewing port. This port contained a quartz glass window measuring 25 mm in diameter with a thickness of 3.2 mm; the transmission spectra of this window were included in the IRT’s calibration. Positioned 72.5 mm from the combustion chamber, the IRT was calculated to be sighted upon a target area of approximately 24.2 mm in diameter, based upon the IRT’s measured FOV of 3:1. The IRT measured the temperature of the closed combustion chamber over time, with an acquisition time of 1 µs to evaluate the rapid fluctuation in the mean temperature of the chamber. An additional averaged 1 ms measurement was overlaid to define the overall trend of the droplet temperature measurement.

## 3. Results and Discussion

### 3.1. FPGA Characteristics, Implementation and Testing

The FPGA module for the IRT was validated with respect to the compilation summary and estimated device utilisation. These were generated within LabView, achieving a data acquisition time of 1 µs. Device utilisation results were obtained with a 40 MHz [46] onboard clock. Out of 10,250 slices, 3825 were used, with 10,015 out of 41,000 slice LUTs used. The results, calculated using Equations (2) and (3), yielded a throughput of 0.64 Gbps and efficiencies of 0.17 Mbps/slice and 0.062 Mbps/slice in LUTs, respectively. This confirms a low-area design in comparison to a conventional FPGA, where similar designs described in [47,48] report similar performance in efficiency and throughput with higher clock frequencies ranging between 0.3 Gbps (91.6 MHz) and 1.3 Gbps (425 MHz).

**Figure 3:**
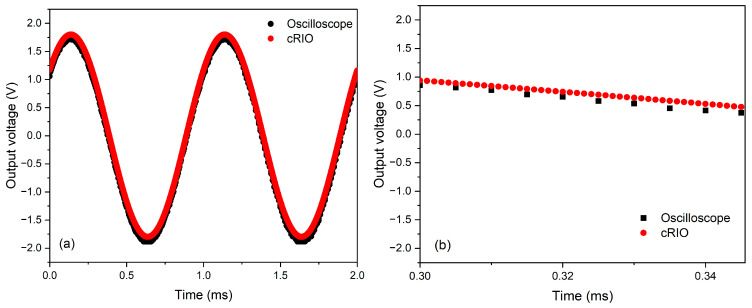
Response time analysis of the (**a**) oscilloscope with respect to the (**b**) cRIO over a sampling time of 5 μs and 1 μs, respectively, with a 1 kHz sine wave.

The data acquisition time of the cRIO, in relation to the IRT, was verified, using a 1 kHz sinusoidal signal from the waveform generator, as shown in Figure 3a. The cRIO acquires the received signal at a 1:5 sampling time ratio compared to the 5 μs acquisition time of the oscilloscope. This confirms that the cRIO is correctly logging at 1 μs, as depicted in Figure 3b.

### 3.2. IRT Performance Evaluation and Characterisation

**Figure 4:**
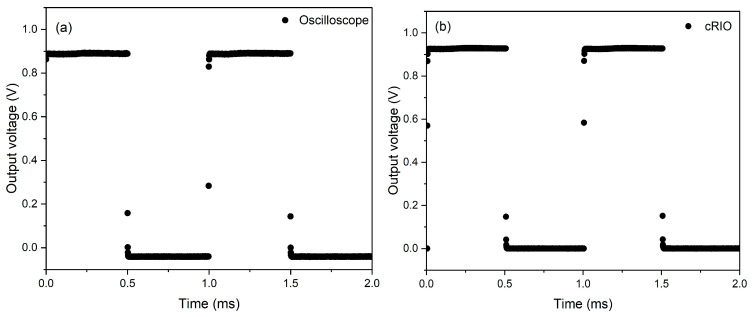
Response time assessment of the IRT with a 1 kHz pulsed square wave with an 870 nm LED recorded from (**a**) the oscilloscope and (**b**) the cRIO.

To radiometrically validate the use of the IRT for 1 μs temperature measurements, the IRT was characterised using the various measurement techniques described in Section 2.2. The output voltage of the IRT, in response to an LED pulse with a 1 kHz square wave, is shown in Figure 4, demonstrating the rise time of the IRT analogue electronics and the overall IRT-cRIO measurement system. The response time of the IRT analogue electronics in Figure 4a was found to be less than 900 ns, ensuring its compatibility with the subsequent 1 μs acquisition time of the cRIO. The equivalent measurement is displayed in Figure 4b, where the output of the IRT is connected to the cRIO acquisition system.

**Figure 5:**
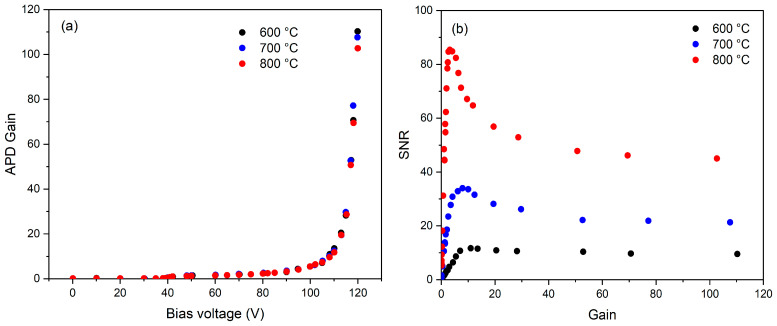
(**a**) APD gain with bias voltage and (**b**) SNR as a function of APD gain at target temperatures of 600 °C, 700 °C, and 800 °C.

The gain of the Si APD-TIA as a function of the bias voltage was measured, as depicted in Figure 5a. To assess this gain and to determine the optimal bias voltage for use within the IRT, we conducted measurements at target temperatures of 600 °C, 700 °C, and 800 °C. The output voltage increased following an increase in the reverse bias voltage, as a result of the increased electric field strength within the APD. The unity gain point was found to be at approximately −42 V, with all other gains determined in reference to this point. The gain was found to be consistent across all target temperatures measured, indicating wavelength independence in the gain over this target temperature region.

The SNR of the APD-IRT was calculated as a function of gain across these target temperatures to establish the optimum gain of the APD-IRT, as shown in Figure 5b. The SNR with respect to the gain increases up to a maximum point before decreasing again for each of these target temperatures. Beyond this maximum point, the excess noise within the APD starts to dominate the amplifier noise, resulting in a drop in the overall SNR. This implies that the optimal operational gain and corresponding reverse bias voltage of the APD need to align with its maximum SNR value at the minimum target temperature that the instrument is calibrated to measure, which, in this work, is 600 °C. This equates to an approximate APD gain of 11 for our APD-IRT, with this gain maintained across the full dynamic range of the target temperatures measured.

**Figure 6:**
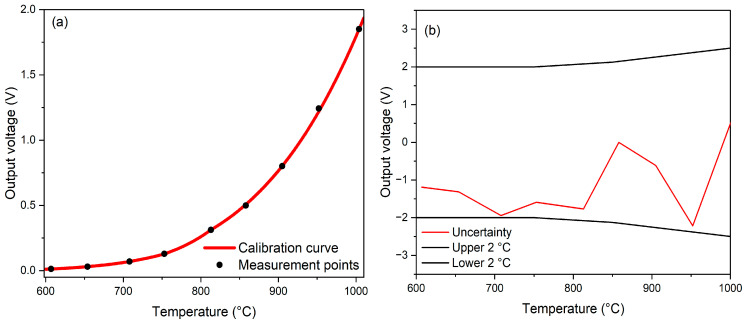
(**a**) Output voltage as a function of temperatures and the (**b**) uncertainty quantification over the target temperatures.

The mean output voltage of the IRT, as a function of target temperature between 600 °C and 1000 °C, is shown in Figure 6a, indicating that both the measured and calibrated output voltages demonstrate adherence to Planck’s law. The resultant calibration uncertainty curve is presented in Figure 6b, with calibration performed at target temperatures of 607 °C, 753 °C, 858 °C, 905 °C, and 1004 °C. The measured uncertainty of the IRT was found to be within ±0.25% °C + 2 °C, showing that our IRT lies within the uncertainty bounds of typical commercial instruments.

**Figure 7:**
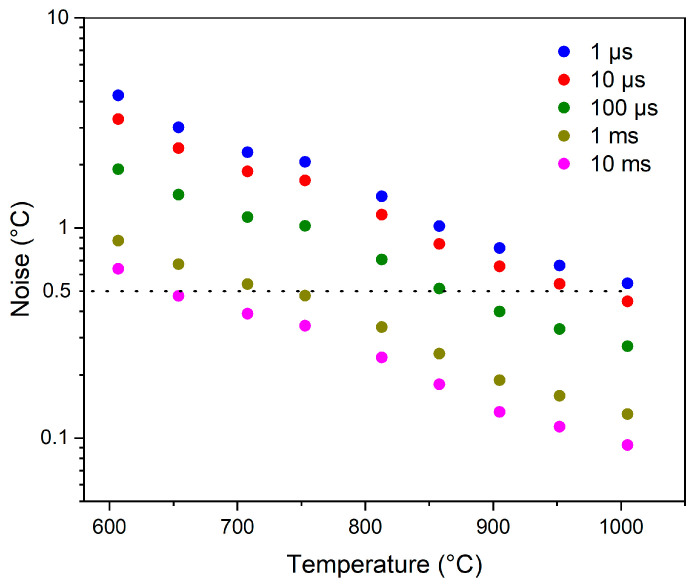
RMS noise as a function of target temperature for integration times between 1 μs and 10 ms.

Evaluated over integration times ranging from 1 μs to 10 ms, the RMS noise performance of the IRT is shown in Figure 7 as a function of target temperature between 600 °C and 1000 °C. For reference, the typical commercial noise specification of 0.5 °C has also been included and is represented by the dotted horizontal line. The IRT achieves this noise specification with an acquisition time of 1 µs at a target temperature of approximately 1000 °C. Whilst this minimum temperature to satisfy the 0.5 °C noise specification is higher than that of commercial 1 µm IRTs, it is important to note that the response time of our instrument is significantly faster. An improvement in the RMS noise performance can be observed with increased integration time. This trend signifies an increase in the minimum target temperature to satisfy the 0.5 °C noise specification to temperatures of less than 950 °C and 850 °C for integration times of 10 µs and 100 µs, respectively. Furthermore, for integration times of greater than 1 ms, the noise specification is met at 750 °C and 650 °C, respectively. Therefore, when compared to typical commercial instruments, the noise performance of our IRT aligns well, indicating that the noise performance at 1 µs is reasonable and within expectations for such a high-speed instrument.

### 3.3. Closed Combustion Temperature Measurement

The fast response time and noise performance of our instrument validate its use within high-speed non-contact temperature measurement applications. This instrument is therefore ideally suited to measure rapid temperature changes and provide additional insights into the early stages of a burning droplet within a sealed combustion chamber. The high sampling rate of the IRT, which enables a greater level of temporal precision, has the potential to reveal more of what may be happening during the initial stages of such events. Furthermore, by incorporating averages of the measured temperature of the combustion chamber, the IRT enables us to better understand the overall trend in temperature during the combustion process of aviation fuels.

**Figure 8:**
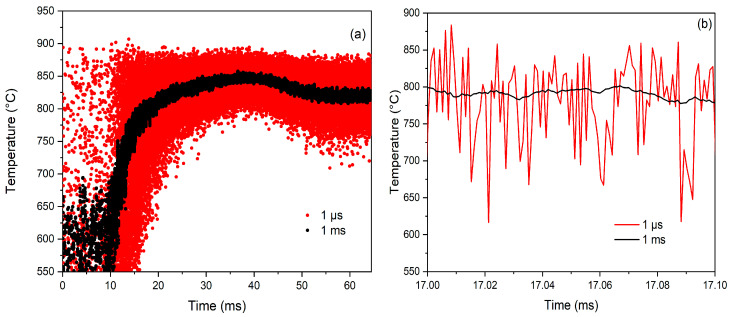
(**a**) Analysis of the mean system temperature of the combustion chamber and (**b**) the rapid transient response during ignition.

The IRT was positioned within the measurement setup, as shown in Figure 2, and a droplet of standard aviation jet A kerosene fuel was subsequently ignited. Each 1 µs spaced dot within Figure 8a represents a snapshot in time of the mean temperature of the system at a specific moment during the droplet combustion process. In contrast, the overlaid averaged 1 ms measurement shows the overall trend observed in the average temperature, spanning various stages from 0 ms to 65 ms.

From 0 ms to 10 ms, the scattered data points, which can be visualised as irregularities in the temperature trend, can be attributed to the high energy released by the electric spark to start the ignition process. The resultant high energy release from the spark causes a rapid increase in the chamber temperature, which can be observed from 10 ms to 20 ms. This can be attributed to the sudden physical changes caused by the rapid heating of the spark, which includes the boiling and the evaporation of the droplet. From 20 ms to 40 ms, there is a visible reduction in the increase in chamber temperature, implying that the temperature of the droplet is reaching its peak. The flame establishment and the presence of the electric spark lead to this smaller increase in temperature, concluding that the flame and spark are both present during this time interval. After the spark is extinguished, a slight drop in temperature can be observed between 40 ms and 50 ms. From the 50 ms point, the graph shows a steady, flat trend in temperature, indicating the commencement of the self-sustained combustion phase. This means that the combustion process continues independently without the need for an external ignition source; the heat from the combustion itself is sufficient to ignite the fuel–air mixture. This reignition process only terminates once all the fuel is consumed. The trend from the start of ignition until sustained combustion aligns well with what has been reported in the literature [49,50]. This also demonstrates that the temperature of the spark influences the mean system temperature until the spark is extinguished. Given the fast data acquisition speed, the higher temporal resolution of this measurement allows us to observe the individual transitions in temperature and the influence of the spark during the combustion process, thus showing the mean system temperature at each stage of the combustion.

Combustion processes, which are exothermic reactions, can be compared to an electron’s excitation process [51]. These observed variations provide us with the opportunity to perform a detailed examination of the transient response graph, shown in Figure 8b, between 17 ms and 17.10 ms. This analysis not only confirms that the temperature within the enclosed combustion chamber fluctuates but also allows us to observe the instabilities occurring during the initial phases of the ignition process within the closed combustion chamber. The combustion instabilities that are present are physical phenomena occurring in a reacting flow, such as the temperature of the flame, in which some perturbations, even very small ones, grow and then become large enough to alter the features of the flow in some way. As shown in Figure 8b, the temperature fluctuates between 600 °C and 900 °C, resulting in a temperature disparity that can affect the ignition process and lead to delayed combustion, which is not efficient and was only able to be captured by our inherently fast IRT.

These temperature fluctuations not only underscore the importance of understanding what is happening during each time interval within the combustion but also highlight the potential for optimising combustion processes and improving the efficiency further. The acquisition time of 1 µs broadens the characterisation capabilities within the assessment of the expected mean system temperature, highlighting the flame’s contribution to the rapid temperature variations and enabling a more detailed investigation of high-speed transients during the ignition process’s early stages. The 1 µs measurement, attributable to our instrument’s inherent speed, provides a superior temporal resolution throughout the combustion process, leading to a more precise temperature assessment and the capability to capture rapid temperature fluctuations. Figure 7 confirms the effectiveness of our instrument as the noise is reasonable for measurements up to 1000 °C at 1 µs. This indicates that while instrument noise is present, it is minimal compared to the fluctuations observed within the combustion chamber. Therefore, we can confidently assert that the temperature fluctuations measured in the combustion chamber are real, despite the expected instabilities of the closed system. Our results therefore highlight the importance of developing such high-speed measurement instrumentation for understanding and optimising combustion processes.

These observations are significant for understanding the temperature behaviour of a single-aviation fuel droplet during reignition, as spray combustion is generally difficult to model and analyse. This is particularly important in industrial settings, where precise control over combustion is essential for further understanding the various characteristics and the future utilisation of SAFs. Further exploration under a range of ambient conditions will enhance our rigorous testing regime and deepen our understanding of SAFs in representative environments, facilitating the transition away from fossil-based aviation fuels.

## 4. Conclusions

In this work, a high-speed 1 µm IRT was developed and showcased for the detailed analysis of the mean system temperature within a closed combustion chamber after the ignition of standard aviation jet A kerosene. The high-speed, FPGA-based architecture, complemented by a highly sensitive Si APD, achieved a data acquisition time of 1 µs. This instrument enabled the measurement of temperature at an enhanced sampling rate, offering a refined temporal resolution across the target temperature range of 600 °C to 1000 °C. The instrument was used to measure the first 65 ms of the standard aviation jet A kerosene droplet ignition process, providing a powerful tool for characterising the fluctuations in the mean system temperature during the droplet combustion process. Its higher temporal precision enables the measurement of rapid temperature changes within the combustion chamber, providing insights into the system’s stability at various stages. The capabilities of the instrument mean that this measurement technique will enable such measurements with SAFs as well, providing greater insight and understanding of their properties. The instrumentation therefore holds considerable promise for facilitating high-speed measurements in the aviation industry for the understanding of fuel combustion processes.

## Data Availability

All relevant data are shown in the paper or can be recreated by following the methodology in this paper.
